# Empagliflozin as treatment in glycogen storage disease type IB patients

**DOI:** 10.1016/j.ymgmr.2025.101226

**Published:** 2025-05-03

**Authors:** María Arbelo Rodríguez, Elena Márquez Mesa, Cristina Lorenzo González, Marina Gutiérrez Vilar, Loredana Arhip, Mónica Ruiz Pons, José Pablo Suárez Llanos

**Affiliations:** a Endocrinology and Nutrition Department, Hospital Universitario Nuestra Señora de Candelaria, Ctra. Del Rosario 145, Santa Cruz de Tenerife 38010, Spain; b Pediatrics Department Hospital Universitario Nuestra Señora de Candelaria, Ctra. Del Rosario 145, Santa Cruz de Tenerife 38010, Spain; c Nutricia-Danone, C/De Buenos Aires, 21, L'Eixample, Barcelona 08029, Spain

**Keywords:** Glycogen storage disease type Ib, Empagliflozin, Neutropenia, Neutrophil dysfunction, SGLT2 inhibitors, 1,5-andhydroglucitol, Glucose-6-phosphate transporter

## Abstract

**Background:**

Glycogen Storage Disease Ib (GSD Ib) is a disease that associates both neutropenia and neutrophil dysfunction, thereby causing recurrent infections and inflammatory bowel disease (IBD). As of now, the standard treatment of these complications has been the administration of granulocyte-stimulating factor (GCSF). However, recent studies have found that the use of empagliflozin, an antidiabetic drug, may have benefits by reducing the levels of 1,5 anhydroglucitol-6-phosphate (1,5-AG6P), a metabolite that accumulates in the cytosol of neutrophils and blocks the use of glucose.

**Results:**

We therefore report our experience with three patients, one of them being a liver and kidney transplant recipient, with promising results. Morbidity has been greatly reduced in all cases consisting in weight gain, better neutrophil count and management of respiratory, osteoarticular and gastrointestinal comorbidities. Overall, an improvement in quality of life has been observed.

**Conclusion:**

SGLT2 inhibitors, and specifically empagliflozin offer promising results in improving morbidity and quality of life in patients with GSD Ib. In the cases presented, including a patient with double liver-kidney transplant, a good profile of tolerance, safety and effectiveness has been observed.

**Synopsis:**

Empagliflozin offers promising results in improving morbidity and quality of life in patients with GSD Ib, including the first double organ transplant patient treated with this drug.

## 1 Background

Glycogen storage disease type Ib (GSD Ib) (Orphacode 79,259) is a rare autosomal recessive disease, with an estimated prevalence of 1: 500,000, due to mutations in SLC37A4 encoding the glucose-6-phosphate transporter (G6PT) of the endoplasmic reticulum. A defect in this enzymatic complex affects glucose formation because both glycogenolysis and gluconeogenesis are inhibited, with negative effects on glycemic control [1,2]. Liver failure and the development of hepatocellular carcinoma make these patients potential candidates for liver transplantation. Moreover, if there is not good metabolic control, kidney damage occurs (glomerular dysfunction) that can progress to kidney failure and require a transplant. Neutropenia is the hallmark feature of GSD Ib, however, the age at onset as well as the clinical course are variable. It may be present at birth or not appear until late in childhood as cyclic or permanent neutropenia. Polymorphonuclear neutrophils (PMNs) from patients with GSD Ib are not only reduced in number, but also dysfunctional and this makes individuals prone to recurring infections [3]. Accumulation of 1,5- anhydroglucitol-6-phosphate (1,5-AG6P) within the granulocytes result in their energy deficiency and consequent apoptosis [4]. Plasma 1,5-anhydroglucitol (1,5-AG) is an analog of glucose, and enters cells where it is phosphorylated to 1,5-AG6P.

Sodium glucose co-transporter 2 inhibitors (SGLT2-I) such as empagliflozin are anti-diabetic drugs that inhibit renal glucose reabsorption resulting in an increased urinary excretion of glucose. This process results in the decrease of renal 1,5-AG reabsorption and, thereby, lowering its serum concentration. It has been proved that, in a G6PC3 (glucose-6-phosphatase catalytic subunit 3) deficient mouse model, that phenotypically and biochemically mimics the PMN impairment of patients with GSD Ib, treatment with an SGLT2- I was able to lower the blood level of 1,5-AG6P and consequently restore a normal neutrophil count [4]. Recently, the same result was replicated in two G6PC3- deficient children [5].

For this reason, there is an increasing interest in investigating how the application of SGLT2-I in clinical practice is evolving. We report our experience in managing 3 GSD Ib patients with empagliflozin and discuss the possible benefits and difficulties that it brings in the treatment of this pathology.

## 2 Methods

A case series that include all patients with empagliflozin being initiated as treatment for GDS Ib and follow-up by the Endocrinology Service of the Nuestra Señora de la Candelaria University Hospital (HUSNC), Santa Cruz de Tenerife, Spain.

The information has been collected from the clinical records. The collected data was: epidemiological (age, sex, weight and height), clinical (cardiovascular risk factors, presence of GSD Ib and associated comorbidities), blood work (hemoglobin, neutrophil and calprotectin count), in-hospital (number of admissions and their main cause) and follow-up.

Data collection was compliant with the Law on Protection of Personal Data and Guarantee of Digital Rights (Spanish Organic Law 3/2018). All patients received information about off-label use of the treatment. All three patients signed the informed consent.

## 3 Results

### 3.1 Case report 1

A 19-year-old woman, diagnosed of GSD Ib (1211delCT) in 2004, when she was one month old, after suffering from severe hypoglycemia, hypotonia and hepatomegaly. Dietary treatment was started. After 3 months, she was discharged with a percutaneous endoscopic gastrostomy to ensure the provision of carbohydrates. During the following years, she was hospitalized several times because of nearby skin infection showing concurrent neutropenia in laboratory tests. This motivated the closure of the gastrostomy at 2-years-old, introducing a nasoduodenal tube as the preferred method of feeding. However, the neutrophil dysfunction persists even after ambulatory treatment initiation with granulocyte colony-stimulating factor (GCSF), causing once again the hospitalization of the patient every 2–3 months because of new and varied infectious processes.

When she was 9-years-old, after numerous hospital admissions with gastroenteritis as the main diagnosis, a colonoscopy was performed. The patient received the diagnosis of inflammatory bowel disease (IBD) after the medical procedure showed multiple ulcers from terminal ileum till left colon, starting treatment. The nasoduodenal tube was then also removed after careful total oral tolerance was observed. No further hospitalizations occurred until a septic shock with respiratory origin occurs 4 years later, requiring an intensive care unit admission with catecholamine treatment and mechanical ventilation to maintain hemodynamic stabilization. After the discharge, she continued with assisted ventilation at home in the form of nocturnal continuous positive airway pressure.

After 3 years with no improvement in neutrophil (absolute neutrophil count (ANC) ≤ 0.2–0.5 × 10^9^/L), empagliflozin is initiated in October of 2020, the patient being 16 years old, weighting 45.5 kg with height being 146.2 cm, resulting in a body mass index (BMI) of 21.29 kg/m^2^. She was also treated with long-acting starch 60 g thrice a day complimentary with scheduled dietary advice, enalapril, mesalazine, gabapentine and GCSF 300 μg/day every three weeks. The starting dose of empagliflozin was 10 mg every 24 h for 3 days, and then, it was increased to 10 mg every 12 h indefinitely. The ANC at the start was 0.14 × 10^9^/L, hemoglobin (Hb) 10.5 g/dL, uric acid 6.59 mg/dL and triglycerides (TG) 226 mg/dL.

One month after the start of the treatment, the digestive symptoms disappeared completely, concomitant with a weight gain of 2 kg in a single month and up to 7 kg in total. GCSF was discontinued after 2 years of treatment with the last ANC being of 1.24 × 10^9^/L. Last follow-up with our team was in November 2023 when she presented a weight of 50.3 kg and ANC of 1.32 × 10^9^/L, Hb 14.5 g/dL, uric acid 3.0 mg/dL and TG 256 mg/dL. No new hospital admissions occurred. The nocturnal CPAP was discontinued since February 2023. Lastly, while no endoscopic study has been performed, fecal calprotectin results have stayed positive (>60 μg/g) and varying within a wide range with no apparent clinical repercussion. No side effects have been reported after 3 years of empagliflozin. The patient perceived an overall upgrade in her quality of life following no more hospitalization episodes and especially after being able to stop non-invasive ventilation support.

### 3.2 Case report 2

A 34-year-old woman had severe hypoglycemia, hyperuricemia and hypertriglyceridemia a week after her birth. Imaging tests showed hepatomegaly. She was diagnosed at one month old, with liver biopsy results compatible with GSD Ib (1211delCT).

When she was 26-years-old, after an episode of hypoglycemia during a Rheumatology hospitalization at HUNSC, she began her follow-up in our Endocrinology department. With a history of large joint oligo arthritic crisis resistant to previous analgesic and anti-inflammatory treatment, results were compatible with Sjögren disease and axonal polyneuropathy. She was also suspected of having either IBD or bacterial overgrowth following numerous episodes of diarrhea during the past year. With normal gastro- and colonoscopy, both antibiotic and 5-aminosalicylic acid (ASA-5) were initiated.

She has periodically been evaluated by with both the department of Hematology, who administered GCSF 300 μg/d every 48 h; and the department of Gastroenterology, who closely followed a stable hepatic adenoma, maximum diameter of 2,5 cm. No clear diagnosis of IBD was shown.

Treatment with empagliflozin was started in July 2021, when she was 31-years-old, with an initial dose of 10 mg every 24 h for 7 days and then 10 mg every 12 h. Her pre-treatment anthropometrics were: weight of 74 kg, height 152 cm and BMI of 32 kg/m^2^. Laboratory studies demonstrated ANC of 0.2 × 10^9^/L, Hb 13.5 g/dL, uric acid 7.1 mg/dL and TG 108 mg/dL. She was given dietary advice and was educated about treatment with long-acting starch 60 g four times per day. In January 2024, the weight remained stable as well as the hemoglobin total count (14.2 g/dL now). Last ANC was of 7.16 × 10^9^/L, with the administration of GCSF 300 μg/d every 72 h. We proposed its suspension in the last consultative encounter. Uric acid decreased (3.5 mg/dL) and TG remained stable (165 mg/dL). In addition, she reported a decrease in axonal polyneuropathy symptoms, this being less walking instability.

No new hospital admissions nor infectious processes have been observed since the start of the treatment. No side effects were reported. The improvement of peripheral neuropathy has been noted by the patient as the most favorable change after initiating the treatment.

### 3.3 Case report 3

A 40-year-old male was diagnosed with GSD Ib shortly after his birth in 1983. He received a liver transplant when he was 7-years-old due to recurrent symptomatic hypoglycemia. He underwent several immunosuppression treatment modifications in the following years after showing various side effects (gingival hyperplasia with cyclosporine, severe neutropenia with hemolytic anemia with tacrolimus). However, the immunosuppressant therapy resulted in chronic kidney disease with kidney biopsy compatible with focal and segmental glomerulosclerosis, requiring a kidney transplant at the age of 28-years-old.

The patient was admitted to the intensive care unit, when he was 30-years-old, with diagnosis of community acquired pneumonia (pneumococcus).

In 2016, with 32-years-old, a lymphoproliferative syndrome associated with post-liver transplant herpes virus-4 infection prompts a right hemicolectomy. Three small bowel obstructions occurred as a result of surgical adhesions, two of which required additional surgical procedures during the same year and 2 years later.

The ANC did not improve over time, even after treatment with GCSF. Recurrent pneumonia was the leading cause of hospitalizations in the year 2016 and 2019. In 2018 the patient started having frequent stools, weight loss and consequent hypoalbuminemia. Colonoscopy results were compatible with IBD-like syndrome. It was also in this year that another intensive care unit admission was necessary after a septic shock of urinary tract origin.

At the end of 2021, another hospital admission occurred, with severe neutropenia being the cause of a resistant urinary tract infections.

Treatment with empagliflozin (10 mg every 24 h) was initiated in January 2022. The pretreatment ANC was 0.4 × 10^9^/L despite GCSF 300 μg/day every 72 h. His weight at the time was 45.6 kg, height 160 cm and BMI of 17.06 kg/m^2^. Additionally, the concurrent immunosuppressive treatment consisted in low dose cyclosporine and prednisone. He presented with Hb 13 g/dL, uric acid 8,72 mg/dL and TG 54 mg/dL.

Three months after the start of empagliflozin, the digestive symptoms disappeared completely, which coincided with a weight gain of 3 kg. GCSF was discontinued in December of 2022, with ANC of 2.88 × 10^9^/L. It should be noted that the total empagliflozin dose was increased in the following month to 10 mg every 12 h, however, poor therapeutic adherence was noted. Constipation for the past 3 weeks after the start of empagliflozin was reported as the cause for poor compliance, with complete resolution after a reduction dose of 10 mg every 24 h. In the March 2023 visit, laboratory values included ANC 4.33 × 10^9^/L, Hb 14.5 g/dL, uric acid 5.24 mg/dL and TG 56 mg/dL. Additionally, the patient has been evaluated twice in the emergency room, in August and September 2023. In August, he was diagnosed with an upper respiratory tract infection. After careful evaluation, he was discharged and started a short course of antibiotic monotherapy, which led to a complete resolution of the symptoms. In September, the patient reported dysuria for the past few days. He was discharged after normal results in both blood and urine samples. No other side effects were reported. As of 2024, up to 6 kg have been gained, and he has not required new admissions. Taking all of this into account, alongside the aforementioned cease of gastrointestinal symptoms, the patient experienced a significant improvement in his quality of life: a less hospital-dependent lifestyle appeared possible, the resumption of frequently social interactions and being able to start exercising.

## 4 Discussion

SGLT2-I are glucosuric drugs implemented as one of the fundamental pillars of treatment for Type 2 Diabetes Mellitus. Not only have they demonstrated benefit in this field, but their use has also been expanded in the treatment of symptomatic chronic heart failure and in chronic kidney disease [6,7].

The previously presented cases outlined empagliflozin as a promising treatment for neutropenia in patients with GSD Ib. In our cases, an initial dose of 10 mg of empagliflozin was chosen, with subsequent progression to a dose of 10 mg every 12 h. If we compare it with the weight of each of them, we obtain weight-adjusted doses of approximately 0.4 mg/kg, 0.3 mg/kg and 0.4 mg/kg respectively. This is in line with previous studies conducted with empagliflozin that used height-weight-adjusted doses between 0.3 and 0.7 mg/kg/day [8]. Dose increase was carried out in three days in the case of our first patient, one week in the second patient and one month in the third patient (a slower dose increase was performed due to patient characteristics and comorbidities). No differences were observed in the tolerance of the treatment. Likewise, other authors have evaluated dose escalation at different speeds without reporting differences on its tolerance, from 2 days [9], 7 days [10,11], 10 days [12], up to several weeks [13,14]. On the other hand, the administration of this drug has been carried out twice daily, every 12 h, at half the total dose. This is due to the inconsistency in the intensity of glycosuria in a 24-h period, therefore, reducing the risk of hypoglycemia that we would obtain with high doses [13]. However, it should be noted that although this has been the method of choice we have elected, other authors have chosen to administer a single dose [9]. Similar results have been described in both treatment regimens [15]. These findings, at first contradictory, may be due to the almost equal total dose used adjusted for weight.

**Fig. 1:**
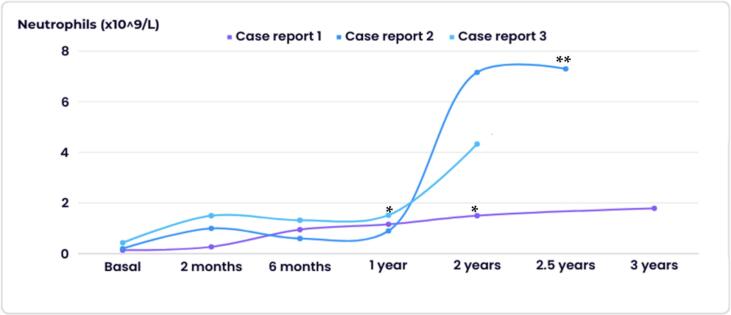
Evolution of ANC after treatment with empagliflozin. * GCSF was discontinued. ** GCSF was proposed for suspension.

In terms of effectiveness, improvement in ANC has been achieved in all our patients. After the initiation of treatment, the clinical and analytical improvement is clear and consistent with current evidence [[8], [9], [10], [11], [12], [13], [14], [15]]. Furthermore, all three patients have shown an increase in ANC even after 12 months, as illustrated in Fig. 1. While not widely recognized, there have been some reports confirming the stabilization and enhancement of neutrophil function over extended periods of time [9,16]. (See Table 1.)

**Table 1:** Empagliflozin effects in GSD Ib patients.

Gender	Age	Empagliflozin final dosis	Treatment duration	Neutrophil count	IBD	Hypoglycemic events	Uric acid	TG	Other treatments	Notes
♀	19 y	0.4 mg/kg/d	3 y	↑ Stop G-CSF	↓	None	↓	↔	enalapril mesalazine gabapentine	Mechanic ventilation (stop)
♀	33 y	0.3 mg/kg/d	2.5 y	↑ ↓ G-CSF	N/A	None	↓	↔	enalapril febuxostat rituximab	Sjögren disease Axonal polyneuropathy ↓ Hepatic adenoma ↔
♂	40 y	0.4/mg/kg/d	2 y	↑ Stop G-CSF	↓	None	↓	↔	enalapril febuxostat cyclosporine prednisone	Liver transplant Kidney transplant

TG, triglycerides; IBD, inflammatory bowel disease; y, years; ↑, increase; ↓, decrease; ↔, stable; N/A, not applicable.

We do not have the necessary means in our center to evaluate white cell quality. Recent studies suggest that this clinical improvement in neutropenia does not have to correlate with a clear increase in the absolute neutrophil count, highlighting the relationship between symptomatic improvement and the decrease in 1,5AG6P in neutrophils [[8], [9], [10], [11], [12]]. An inverse correlation has been noted between 1,5AG concentration and the neutrophil count, chemotactic response, phagocytosis and NETosis [17]. The reduction in infections in these patients is not solely due to an increase in neutrophil count they also enhance their functional capacity, which is crucial for an effective immune response. This functional improvement is key to the reduction in infections, as evidenced by the results in our patients, who showed a lower frequency of infections following treatment. Moreover, a direct consequence of increased numbers and probably increased functionality of neutrophils is reflected in the decreased GCSF needs in our patients. In 2 out of 3 patients it has been completely removed, with good response. At the same time, withdrawal of treatment has been considered in the remaining patient. It is observed that there is a tendency towards complete discontinuation of GCSF in the literature [[10], [11], [12], [13], [14], [15],^18^]. Poor response to its withdrawal has been reported only once, in the end maintaining treatment with GCSF at doses significantly lower than those required prior to starting empagliflozin [13]. Regarding Hb levels, we have observed a significant improvement in the first patient, slight improvement in the third patient and remaining stable in second one. In previous studies, a trend towards resolution of anemia has been observed [13].

There is a fair amount of data on IBD improvement in GSD Ib patients treated with empagliflozin. A cohort of 66 GSD Ib individuals with IBD prior to drug exposure was analyzed. Out of a total population of 110 individuals, 40 % did not present clinical findings of IBD before the drug treatment, while 78 % showed no IBD findings during the drug exposure [15]. In a small pediatric sample, clinical effectiveness rate of 83.3 % has been reported [19].

In our sample, both patient number 1 and patient number 3 showed rapid improvement of gastrointestinal symptoms as well as weight gain after empagliflozin initiation. On the other hand, patient number 2 had good clinical control prior to the start of the treatment. However, all this information regarding the improvement of the disease is limited to the clinical aspect. No follow-up with imaging tests or scales have been performed in these patients. The current evidence, although limited, indicates how interesting it would be to evaluate the evolution of IBD with biopsy or colonoscopy [11]. Despite this, the improvement in the quality of life of these patients is evident [12,13,18].

In the context of metabolic alterations of GSD Ib, patients can manifest hyperuricemia and hypertriglyceridemia. Although only a few authors reported pre-post SGLT2-I values of uric acid and some cases were concurrently on allopurinol to prevent renal damage [13], empagliflozin seems to have ameliorative effects on hyperuricemia in some GSD Ib patients [[11], [12], [13]]. In our cases, all patients had a reduction in uric acid levels. This might be reflective of improved metabolic control but it is not clear if it could be directly related to the levels of 1,5-AG [20]. TG values were reported to have a variable trend during therapy [13,19], in our patients the values remained stable.

An intriguing finding in our cohort was the reported improvement of axonal polyneuropathy symptoms in the second patient, which was described as greater stability when walking. Although the mechanisms remain speculative, this observation aligns with evidence demonstrating the neuroprotective effects of empagliflozin in diabetic rat models [2, [21]. These studies suggest that empagliflozin may improve nerve conduction and morphology through AMPK pathway activation, reduction of oxidative stress, modulation of inflammatory cytokines and enhancement of autophagy via mTOR inhibition. Furthermore, clinical data in humans have shown that, after three months of treatment, patients receiving empagliflozin exhibited significant improvements in electrophysiological studies and a reduction in biomarkers of oxidative stress and neuronal damage (NSE and MDA), despite no significant changes in glycemic control or cardiovascular risk scores [22]. Given that the patient's neuropathy may have had an immune-mediated component, possibly related to Sjögren's syndrome, it is conceivable that empagliflozin's anti-inflammatory and immunomodulatory effects contributed to the improvement observed.

Liver transplantation in GSD Ib has been indicated for severe glucose intolerance, poor metabolic control and poor growth [23], however, reports are scarce. We have not found any published evidence regarding GSD Ib and kidney transplant. In this context, it is important to consider evidence of treatment with empagliflozin in GSD Ib organ transplant recipients is limited. A recent international consensus recommends continuing empagliflozin after liver transplantation, even if the exact moment of restarting it needs to be individualized [24]. On the other hand, two GSD Ib patients who received liver transplantation and treatment with SGLT2-I have been reported [25]. One of them started empagliflozin after the first transplantation, while the other one started it before the procedure. No side effects were observed, while increase of PMN count and decrease of 1,5-AG levels were detected. At the same time, the administration of empagliflozin or any other SGLT2-I in kidney and liver transplant recipients is very limited.

An increasing number of articles in recent years present promising results through the use of empagliflozin in kidney transplant recipients with diabetes mellitus, pre- and post-transplant [[26], [27], [28], [29]]. The EMPA-Renal Tx 2019 found that SGLT2 inhibitors likely have no effect on kidney graft survival and does not increase hypoglycemia. However, eGFR transiently decreased at 8 weeks, and urinary tract infections were a concern [27]. Nevertheless, other studies [26,[29], [30]] did not increase urinary tract infections and did not harm kidney function [26,30].

The main results seem similar to those of our patient. Kidney function remains stable, sometimes even improving after the initiation of treatment, or with slight initial deterioration. On the other hand, in regard of the safety of starting glucosuric treatment with a known relative frequent adverse effect of urinary tract infection on immunosuppressed patients (including pyelonephritis and urosepsis); in our case, this side effect has not been observed to date. Although the risk exists [28], the increased severity of the infection in this group of patients and its causal relationship with the drug is controversial. The long-term immunosuppression required after transplantation predisposes patients to opportunistic infections. Neutrophil function gain could avoid the increase of infectious processes in these patients. While there is no direct evidence to date, it is noteworthy to consider whether the potential role of empagliflozin in neutrophil function could potentially contribute to better post-transplant outcomes. Notably, no transplant rejection was observed in our case.

In terms of metabolic control, no increase in hypoglycemic episodes was observed in any of the 3 patients. As has been established, the primary metabolic abnormality of GSD Ib is fasting hypoglycemia [31]. A recent retrospective cohort study of patients with GSD Ib, including both pediatric and adult population, reports fasting hypoglycemia in all 113 patients [32]. In this regard, in a cohort of 14 patients with GSD I (2 GSD Ib) CGM revealed periods of low blood glucose below 72 mg/dl in all patients [33].

Large sample sizes effectively highlight an increase in hypoglycemia episodes after empagliflozin initiation. Level three hypoglycemia (< 54 mg/dl) was the most common adverse effect reported (18 % of all patients) [15]. Along these lines, 50 % of Halligan et al. cohort of 8 GSD Ib patients showed hypoglycemic episodes [13]. However, they have not been able to determine whether they are a consequence of the drug, the underlying disease [15] or a carbohydrate deficit in the diet [13].

The third patient presented has undergone both renal and hepatic transplants. Interactions may exist between commonly used immunosuppressants and SGLT2 inhibitors, however, the view is that potential interactions are overall modest and unlikely to result in hypoglycemia or compromise immunosuppression [34]. For example, treatment with canagliflozin has been documented as safe with no significant change in episodes of hypoglycemia [35]. One study emphasizes subjective improvement in severity of hypoglycemic events due to decrease in blood glucose lability [36].

Other side effects have not been observed in direct relation to the initiation of empagliflozin. One of our patients reported constipation, consistent with findings reported in other studies [13]. However, poor therapeutic adherence at this moment prevents us from correlating the symptoms described with the drug. Other studies also do not report adverse effects with empagliflozin [18], or minor side effects with resolution after dose reduction [12].

Furthermore, it has recently been published that treatment with empagliflozin in patients with GSD Ib is associated with a significant reduction in costs for the healthcare system [37].

This case series has several limitations. Firstly, our results were obtained with a treatment used with an off-label indication and from a single hospital, with a very limited sample of a very rare disease. Secondly, the follow-up of our patients is limited. Patients are referred to our consultation as adults and, at the same time, as a particularity of our health area one of our patients has a medical history in different hospitals. Currently we do not have a quick and direct platform that allows sharing medical reports between the area. Thirdly, the approach to the disease is multidisciplinary. It would be interesting to evaluate in future studies the findings jointly with other departments involved in the management of the disease, with the relevant tests required. Fourthly, currently we are not able to determine 1,5-AG concentration in blood due to technological limitations. Finally, even though the three cases during consultation described satisfaction with the SGLT2-I treatment, referred as both the reduction of limiting symptomatology and either reduce or removal of other, more invasive, treatments; no quality of life specific questionnaires or scales were implemented.

Expanded studies with larger samples, more years of follow-up and in multiple hospital settings are required to confirm the results observed in our case series.

The strength of our work was that we included a kidney and liver transplant patient who demonstrated a good clinical response with improvement in ANC, resolution of digestive symptoms and infectious complications without adverse side effects.

## 5 Conclusion

Empagliflozin offers promising results in improving morbidity and quality of life in patients with GSD Ib. In the 3 cases presented, a good profile of tolerance, safety and effectiveness of empagliflozin has been observed. However, expanded studies with larger samples, more years of follow-up and in multiple hospital settings are required to confirm the results observed in our case series. Additionally, it is essential to recognize the need for more standardized reporting. as current evidence is largely limited to individual case reports. An international registry for patients receiving SGLT2 inhibitors could help generate more cohesive data and support multicenter trials with defined efficacy endpoints. This would be especially valuable given the off-label use of these agents, providing robust data to inform potential future approvals.”

## Availability of data and materials

Not applicable.

## CRediT authorship contribution statement

**María Arbelo Rodríguez:** Writing – review & editing, Writing – original draft, Visualization, Validation, Resources, Methodology, Investigation, Conceptualization. **Elena Márquez Mesa:** Writing – review & editing, Writing – original draft, Validation, Resources, Project administration, Methodology, Investigation, Conceptualization. **Cristina Lorenzo González:** Writing – original draft, Visualization. **Marina Gutiérrez Vilar:** Writing – original draft. **Loredana Arhip:** Writing – original draft, Visualization. **Mónica Ruiz Pons:** Validation, Supervision, Resources, Project administration, Methodology, Investigation, Conceptualization. **José Pablo Suárez Llanos:** Supervision, Project administration.

## Consent for publication

All patients provided written consent for publication.

## Ethics approval and consent to participate

Not applicable.

## Funding

This research received no external funding.

## Declaration of competing interest

The authors declare they have no competing interests.

## Data Availability

No data was used for the research described in the article.
